# Book Review

**Published:** 1906-12

**Authors:** 


**Green's Encyclopedia and Dictionary of Medicine and Surgery** Vol. I. AAC—BRA—Aachen to Brain. Pp. xii., 538. Edinburgh and London: Wm. Green and Sons.—In this work the attempt is made by the editor to combine the advantages of the *Encyclopedia of Medicine* with those of the *Medical Dictionary.* On the one hand, the reader will find the elaborate articles of the *Encyclopædia Medica* (1899–1904); on the other hand, he will discover a large number of new paragraphs, varying from a few words up to thirty lines, representing the lexicographic portion. The editor (Dr. J. W. Ballantyne) has made himself responsible for the whole of the dictionarial part of the work: the longer articles are written by the most eminent specialists and authorities to be found in the British Islands. The printing of this volume is exceedingly well done, and its value is so obvious that it needs no comment. We trust that the publishers will be rewarded for their enterprise, and that *Green's Dictionary* will be looked upon as a necessary part of the furniture of the consulting-room.

**System of Medicine.** By Many Writers. Edited by THOMAS CLIFFORD ALLBUTT, M.A., M.D., LL.D., and HUMPHRY DAVY ROLLESTON, M.A., M.D. Second Edition. Vol. I. Pp. xvi., 1209. London: Macmillan and Co. Limited. 1905.—We heartily welcome a second edition of this well-known work. One new volume is to appear each year, so that the old edition will be gradually superseded. There are many alterations and additions in the first volume. Among the additions we may mention articles on the "History of Medicine," by Professor Allbutt and Dr. Payne, "Old Age," "Physical Exercises," and "X-Rays in Medicine." The article on the "Clinical Examination of the Blood" has been transferred to this volume from Volume V. Many of the articles have been entirely re-written, including those on the "General Pathology of New Growths," "Medical Statistics," and

"Medical Geography"; while the article on the "General Pathology of Nutrition" has been much enlarged. It is quite unnecessary to insist on the great value and excellence of the articles.

**Heart Disease and Aneurism of the Aorta, with special reference to Prognosis and Treatment.** By Sir William H. Broadbent, Bart., K.C.V.O., F.R.S., and John F. H. Broadbent, M.A., M.D. Fourth Edition. Pp. xvi., 479. London: Baillière, Tindall and Cox. 1906.—A fourth edition of this book requires little comment or commendation. Its preparation has been entrusted to Dr. John F. Broadbent, who has re-arranged the subject-matter and added chapters on the pulse, diseases of the coronary arteries, bradycardia, and atheroma of the aorta. The third edition was reviewed by us in 1900 (vol. xviii., p. 159). It was there mentioned that few physicians have had the opportunities of Sir William Broadbent for forming opinions on the prognosis of various forms of heart disease, and hence this book may be expected to contain the condensed views of a clinical physician of vast experience and of matured judgment. The various chapters on prognosis are well worthy of careful study, and cannot fail to be instructive to those of considerable experience who can best appreciate the views of the writer. The chapter on affections of the myocardium introduces points which are easily overlooked and forgotten except by the specialist. It is there stated that "syphilitic affections of the heart wall are not very common, but are doubtless frequently overlooked." The chapters on fatty degeneration, on angina, on tachycardia, and on bradycardia, are all most instructive. The subjects of acute and chronic degenerative lesions of the aorta lead up to the final chapter on aneurysm of the arch of the aorta. Here the author does not encourage the various modern methods of treatment by injection of gelatine and by electricity; he mentions with favour the administration of chloride or lactate of calcium in doses of 10 to 15 grains two or three times a day, but he clearly trusts far more to the older practice of limiting the food and fluids with the administration of full doses of iodide of potassium.

**Consumption: Treatment at Home and Rules for Living.** By H. Warren Crowe, M.D. Pp. 32. Bristol: John Wright and Co. 1906.—This little book can hardly fail to be of value, not only to the general practitioner, but to all who are interested in the nursing of consumptives in their own homes. Its chief value lies in the fact that it is obviously written by one who has encountered the difficulties attendant on the general practitioner who attempts to enforce the necessary discipline and régime which are of such importance in the life of a consumptive. It is clear that Dr. Warren Crowe has experienced the difficulty of getting consumptives to go to sanatoria, where this régime can best be taught; and since this difficulty so often arises, we can think of no better *vade mecum* for this class of patient.

**The "Nauheim" Treatment of Chronic Diseases of the Heart.** By LESLIE THORNE-THORNE, M.D. Second Edition. Pp. x., 75. London: Baillière, Tindall & Cox. 1906.—This book gives a good practical description of the methods of administration of Nauheim baths, and also of the Schott exercises. The movements of the latter are well illustrated by twenty-eight photographs, while a chapter is devoted to the selection of cases suitable for the treatment. The book forms a useful guide to all the details necessary for the carrying out of the treatment.

**The Consumptive Working Man: What can Sanatoria do for him?** By NOEL DEAN BARDSWELL, M.D. Pp. vii., 202. London: The Scientific Press Limited. 1906.—So much literature has appeared on the subject of tuberculosis during the last few years, that it is with pleasure we record the publication of a practical little volume dealing with a portion of the subject which is receiving much attention abroad at present, and which has been subject to so much controversy in this country. Written as it is by one who has had actual experience in treating the consumptive working class under various conditions, the subject is dealt with both practically and sympathetically, and much valuable information is imparted as to that most important aspect of the question, the after-care of patients. It is clear that unless employers are approached with a view to improved conditions of work after discharge, patients must return to surroundings which are at least in most cases the pre-disposing factor in the acquirement of the disease. But the question bristles with difficulties, and members of health committees, besides the medical profession, will find useful suggestions on this matter. Dr. Bardswell says, "Fortunately for consumptives, ideal climatic conditions are not essential to their treatment," with which we thoroughly concur. Treatment is of primary importance. Life histories occupy much space, and might have been curtailed to advantage. The value of the book is enhanced by an introductory chapter by Sir William Broadbent; whilst the three appendices, especially that dealing with the statistics of German sanatoria for the working classes, will well repay perusal. We regret that nothing is said with regard to the employment of consumptives whilst undergoing treatment, for to our mind this is a valuable means of arriving at some estimate of capability for work on their discharge from sanatoria. We can thoroughly recommend this little volume to all those interested in the subject as an earnest and careful contribution to the literature on the subject.

**The Medical Annual for 1906.** Bristol: John Wright and Co.—The total destruction by fire of the large portion of this

24th issue gave the publishers exceptional difficulties, which have been surmounted nobly, and the volume appears to have lost nothing by the great difficulties of its early life. No previous issue has contained more valuable matter both as regards text and illustrations. We notice that the names of Dr. Watson Williams and Mr. Harold Mole are included in the list of contributors. The volume contains, as usual, much valuable information on every conceivable variety of topic, and should be at hand on the table of the busy practitioner for constant perusal and consultation when in any difficulty.

**Hints on the Management of the Commoner Infections.** By R. W. Marsden, M.D. Pp. viii., 128. London: William Heinemann. 1906.—This book endeavours to present a *résumé* of the principles of treatment to be observed in the management of those commonly occurring infections or intoxications which are due to the direct or indirect action of micro-organisms. The subject is, however, limited by the exclusion of those diseases which are not endemic in this country. The busy practitioner will here find an outline of the practical details of the individual treatment of patients which are of the greatest importance, but which are in the text-books commonly compressed into only a few sentences. From the late medical superintendent of the Monsall Fever Hospital and clinical lecturer in infectious diseases at the Owens College, Manchester, may be expected most authoritative views on all doubtful points in the subjects relating to the invasion of the body by septic micro-organisms.

**Transactions of the College of Physicians of Philadelphia.** Vol. XXVII. Philadelphia: Printed for the College. 1905.—A series of papers forming what is called an X-ray Symposium are an important part of this volume. Dr. Ennion G. Williams concludes "that we have in the X-ray an agent that has a specific and destructive effect upon malignant tumours by taking away the life of their cells, and that its application in treatment is limited only by the ability to reach the growths with the proper amount of energy and safety to the surrounding parts." In the discussion which followed these papers Dr. Johnston (Pittsburg) said that he had demonstrated to his own satisfaction that the X-ray has the power of inhibiting the growth of malignant tissue. "If it has that power, and if the inhibition be continued long enough, we may hope that the malignant tissue may settle down and forget to be an anarchist." Dr. Leonard closed his remarks by saying that "if there is any agent capable of inhibiting the primary growth as well as the metastasis that agent needs to be studied carefully, not only by X-ray men but by pathologists and general practitioners." This is precisely what our X-ray societies are endeavouring to do. The subject of X-ray diagnosis of gastroptosis by the assistance of large doses of bismuth subnitrate is dealt with by Dr. Pfahler.

**Appendicitis: its Pathology and Surgery.** By C. B. Lockwood, F.R.C.S. Second Edition. Pp. xiv., 342. London: Macmillan and Co. Limited. 1906.—The first edition of this work was reviewed in this *Journal* some years ago, and the general plan and arrangement of the first edition has been retained, but new observations have been added and many facts from recent contributions on appendicitis have been incorporated in the new edition. The author says: "The subject is not yet ripe for more general treatment." It is not quite easy to understand this. A very large amount of experience has now been gained, and a vast amount of literature on the subject has accumulated. No doubt the fact that the book is nearly altogether a record of the author's own observation and experience gives it a special value. Many will be curious to know what is the author's present view as to the indications for operation in appendicitis, for many surgeons have considerably altered their practice since the first edition of the book six years ago. Mr. Lockwood does not go so far as to say that it is advisable to operate on all cases. He strongly objects to Sir Frederick Treves's statement that operation is seldom required before the fifth day, for, as he says, some cases of gangrene and of perforation run their course with frightful rapidity, and are soon beyond the reach of surgery. As he says: "The question of operation would cease to be difficult if we could infer the pathological condition of the appendix and of the structures in its vicinity. . . . At the present time our diagnosis does not justify a precise diagnosis in every case." This is just why some of us think it is safer to remove the appendix in all cases. Mr. Lockwood says he has never regretted operating in appendicitis under acute symptoms, and we would suggest that it would be much safer to operate than to watch an acute case "hour by hour" for indications for operation, and then very likely regret when we do operate that those hours have been lost. The chapter on "difficulties and errors" in diagnosis is one of particular value, for we learn much by the mistakes of others—almost as much as by our own. Altogether we can as strongly recommend the careful study of Mr. Lockwood's valuable work as we did in the original review.

**The Urethrotomies and Kidney Capsulotomy.** By Reginald Harrison, F.R.C.S. Pp. 96. London: John Bale, Sons, and Danielsson, Ltd. 1906.—The greater part of this volume contains the substance of the author's recent clinics at the London Medical Graduates' College and Polyclinic. Kidney capsulotomy is dealt with in the last eighteen pages, and there the author reiterates his well-known views on the application of this procedure for the relief of renal tension. He quotes the records of four cases in which the operation was performed. Three of these records are taken from the writer's first paper on the subject, and have been before the profession for years.

We wish he could have given us a much larger list of cases, derived from his own experience, to show the constancy with which strikingly beneficial results or the absence of such might be expected from capsulotomy. The fourth case, the one now newly recorded, is interesting from the fact that suppression of urine following internal urethrotomy appeared to be relieved by the operation. The sections devoted to the consideration of the urethrotomies—internal, external, and combined internal and external—and the application of these procedures in the treatment of stricture, urinary fistulæ, false passages, ruptured urethra, and calculus in the bladder and in the prostate gland, are well worth reading. In them the author's matured opinions are given and the perfected technique he now adopts is described.

**Aids to Surgical Diagnosis.** By H. W. CARSON. Pp. vii., 140. London: Baillière, Tindall and Cox. 1906.—This little book is essentially an elaborated tabular statement of surgical and differential diagnosis, and would appear to be of service to the senior student for revising his information just before an examination. The contents are in general up to date and accurate, and there is evidence of acquaintance with the latest literature of the subject. The manual may be recommended for purposes of reference or revision, and has a satisfactory index.

**Studies from the Department of Pathology of the College of the Physicians and Surgeons of Columbia University, New York.** Vol. x.—This volume consists of the reprints of works which were issued from the laboratory of the pathological department of Columbia University in 1904-5. For purposes of reference, it may be stated that the contents consist of papers on the pneumococcus and streptococcus, the dysentery bacillus, the diphtheria group of bacilli, a case of pernicious anæmia, showing a megaloblastic crisis followed by marked improvement, the electric conductivity of the blood during coagulation, chorion-epithelioma, and dry iodine catgut.

**On Professional Education, with Special Reference to Medicine.** By T. CLIFFORD ALLBUTT, M.A., M.D., F.R.S. Pp. vi., 80. London: Macmillan and Co., Limited. 1906.—This report contains an address delivered to the students of King's College, London, and we can only congratulate them on having been privileged to hear it. The medical profession may also be grateful for the occasion which was the cause of its production, and we are not surprised that Professor Allbutt received a general request for its publication. Professor Allbutt is an acknowledged authority on medical education, and in his capacity as Regius Professor at Cambridge has had ample opportunity of judging the finished results of our present system of medical training. What he has to say therefore as to its merits and defects would in any case deserve respectful attention, and in the present instance his views gain all the additional force derived from wisdom, long experience, and mature reflection. Moreover, they are further commended to us by the eloquence with which they are urged. It is impossible here to discuss the numerous questions raised in the course of this address, questions of very real interest and importance to us. Perhaps the two main points for us at present are, first, in how far does our school training succeed in giving a sound, general education, and in what ways can its admitted defects in this respect be remedied; and secondly, how can we best occupy the years strictly devoted to learning our profession? Professor Allbutt has much to say that is of great value on these and similar subjects, and that is deserving of careful consideration by all who are interested in securing for the medical profession an efficient scientific training. We cordially recommend the book to our readers, and we are sure that they will find it most interesting reading.

**Simple Introductory Lessons in Midwifery.** By M. Loane. Pp. viii., 60. London: The Scientific Press, Ltd.—The aim of this little book is good, namely to try and teach the rather uneducated person the meaning of terms used in midwifery. It is little more than a glossary, and as such it is fairly complete. We rather fail to see the use of the introduction of Latin and Greek derivations to a person who has probably not the slightest idea of either language. A great many of the words used are quite unnecessary for such an examination as that of the Midwives' Board. For instance, eutochia, heterogeneous, hydræmia, &c. A chapter of scarcely two small pages on mechanisms is not enough to be of much use even to a beginner.

**Annual Report of the Smithsonian Institution for the Year ending June 30th, 1904.** Washington: Government Printing Office. 1905.—We always look forward with pleasure to the reading of the papers that form the appendix to the Smithsonian Annual Report, and this volume before us contains as usual a series of papers of the greatest interest. The papers cover a large range of subjects, varying from papers on more abstract science, such as the one on "Variations of Specific Gravity," down to popular subjects such as "Pewter and the Revival of its Use," and "Some New Methods of Lighting." Papers on "Morocco" and the "Present Aspects of the Panama Canal" give useful information on subjects which are at present much before the public, and there are a large number of papers on popular scientific subjects, *e.g.* "The House Sparrow," "Bees and Flowers," &c. There are no papers of strictly medical interest in this volume, the nearest approach being one on "Old Age" by Metchnikoff, in which he expounds his theory of the intestinal microbic cause of old-age degeneration.

